# Black Smokers’ Preferences for Features of a Smoking Cessation App: Qualitative Study

**DOI:** 10.2196/43603

**Published:** 2023-05-30

**Authors:** Chineme Enyioha, Larissa M Loufman, Mary E Grewe, Crystal W Cené, Saif Khairat, Adam O Goldstein, Christine E Kistler

**Affiliations:** 1 Department of Family Medicine University of North Carolina at Chapel Hill Chapel Hill, NC United States; 2 Lineberger Comprehensive Cancer Center University of North Carolina at Chapel Hill Chapel Hill, NC United States; 3 The North Carolina Translational and Clinical Sciences Institute University of North Carolina at Chapel Hill Chapel Hill, NC United States; 4 University of California San Diego School of Medicine San Diego, CA United States; 5 School of Nursing University of North Carolina at Chapel Hill Chapel Hill, NC United States; 6 Carolina Health Informatics Program University of North Carolina at Chapel Hill Chapel Hill, NC United States; 7 Cecil G Sheps Center for Health Services Research University of North Carolina at Chapel Hill Chapel Hill, NC United States

**Keywords:** mobile health apps, smoking cessation, Black smokers, smoking, mobile health, intervention, application, development, online research, interview, functionality, social network

## Abstract

**Background:**

Mobile health (mHealth) interventions for smoking cessation have grown extensively over the last few years. Although these interventions improve cessation rates, studies of these interventions consistently lack sufficient Black smokers; hence knowledge of features that make mHealth interventions attractive to Black smokers is limited. Identifying features of mHealth interventions for smoking cessation preferred by Black smokers is critical to developing an intervention that they are likely to use. This may in turn address smoking cessation challenges and barriers to care, which may reduce smoking-related disparities that currently exist.

**Objective:**

This study aims to identify features of mHealth interventions that appeal to Black smokers using an evidence-based app developed by the National Cancer Institute, QuitGuide, as a reference.

**Methods:**

We recruited Black adult smokers from national web-based research panels with a focus on the Southeastern United States. Participants were asked to download and use QuitGuide for at least a week before participation in remote individual interviews. Participants gave their opinions about features of the QuitGuide app and other mHealth apps they may have used in the past and suggestions for future apps.

**Results:**

Of the 18 participants, 78% (n=14) were women, with age ranging from 32 to 65 years. Themes within five major areas relevant for developing a future mHealth smoking cessation app emerged from the individual interviews: (1) content needs including health and financial benefits of quitting, testimonials from individuals who were successful in quitting, and strategies for quitting; (2) format needs such as images, ability to interact with and respond to elements within the app, and links to other helpful resources; (3) functionality including tracking of smoking behavior and symptoms, provision of tailored feedback and reminders to users, and an app that allows for personalization of functions; (4) social network, such as connecting with friends and family through the app, connecting with other users on social media, and connecting with a smoking cessation coach or therapist; and (5) the need for inclusivity for Black individuals, which may be accomplished through the inclusion of smoking-related information and health statistics specific for Black individuals, the inclusion of testimonials from Black celebrities who successfully quit, and the inclusion of cultural relevance in messages contained in the app.

**Conclusions:**

Certain features of mHealth interventions for smoking cessation were highly preferred by Black smokers based on their use of a preexisting mHealth app, QuitGuide. Some of these preferences are similar to those already identified by the general population, whereas preferences for increasing the inclusivity of the app are more specific to Black smokers. These findings can serve as the groundwork for a large-scale experiment to evaluate preferences with a larger sample size and can be applied in developing mHealth apps that Black smokers may be more likely to use.

## Introduction

Although cigarette smoking in the United States continues to decline, disparities in smoking cessation rates and smoking-related health outcomes remain a problem [1]. Currently, Black smokers succeed less often with smoking cessation compared with White smokers despite efforts to improve smoking cessation rates [2,3]. Although Black smokers start smoking later in life [4], smoke fewer cigarettes a day, and are more likely to be nondaily smokers compared with White smokers and smokers from some other ethnic groups, they succeed less often than White smokers in quitting despite having higher quit intentions and quit attempts [3,5,6]. As recently as 2018, the smoking quit ratio for Black smokers was 48%, which is significantly lower than White and Hispanic smokers who have a quit ratio of 64% and 59%, respectively [7].

Compared with other racial or ethnic groups, Black smokers also bear a disproportionate burden of smoking-related health outcomes [5]. Several reasons for these smoking-related health outcome disparities include Black smokers’ higher likelihood of using highly addictive menthol cigarettes, targeted marketing and advertising by the tobacco industry, lower likelihood of screening for tobacco use, and lower likelihood of being offered smoking cessation advice by their health care providers compared with White smokers [8-11]. Studies also show that Black smokers use evidence-based treatment for smoking less often, which may further contribute to lower success rates [12,13]. These barriers call for effective, accessible interventions that will be more inclusive of and appeal directly to Black smokers.

A growing body of literature highlights the role of mobile health (mHealth) apps for smoking cessation and shows some benefits on smoking cessation [14-17]. Black individuals tend to use their phones for a wider array of functions than other racial or ethnic groups [18] and, therefore, stand to significantly benefit from the use of mHealth apps for smoking cessation. These apps may reduce some of the challenges that Black smokers face such as poor access to smoking cessation services and barriers associated with more traditional face-to-face interventions such as scheduling conflicts, transportation, and cost of treatment [19,20]. mHealth apps can also provide additional benefits such as personalization, reminders and tracking, and opportunities to engage within the social networks of people receiving the same intervention [19]. Despite these known benefits, many studies on mHealth app development for smoking cessation had only a few to no Black participants [21-25]. As a result, there is a paucity of information about which features of these apps may appeal to and be effective for Black smokers. This information is vital for the future development of user-centered mobile apps.

mHealth apps must be user-centered; otherwise they will not appeal to or be used by intended users [26]. To date, various studies evaluated user preferences for smoking cessation mHealth apps, but, to our knowledge, none focused on Black adult smokers. The aim of this study was to investigate the preferences of Black smokers for features of mHealth apps for smoking cessation using QuitGuide as a reference. QuitGuide is a free smoking cessation app that was developed for adult smokers by the National Cancer Institute, with its content and features based on a commonly accessed smoking cessation website [27]. We report on the qualitative results of that investigation.

## Methods

### Study Design and Setting

The research team conducted a qualitative study to explore current and former adult Black smokers’ preferences for mHealth app features via 1-on-1, semistructured, in-depth remote interviews. We used 4 recruitment platforms to identify and screen potential participants for eligibility including the University of North Carolina (UNC) “Research for Me” website, UNC’s mass email system, the national ResearchMatch website [28] with a focus on states in the southeastern region of the United States, and the UNC Department of Family Medicine Tobacco Treatment Program. Interested individuals were directed to a web-based Research Electronic Data Capture (REDCap) screening survey to determine eligibility. Recruitment was done over a 3-month period, and potential participants were assessed for eligibility through the REDCap survey. If eligible, the team contacted potential participants and obtained telephone consent. Afterward, participants received an emailed preinterview REDCap survey to collect demographic and health behavior information. Participants were scheduled for their 1-on-1 remote Zoom interview at the time of consent and received instructions on how to download QuitGuide on their personal devices. Participants were asked to explore the app for at least 1 week before their interview. QuitGuide was chosen as a reference because it is a highly established smoking cessation app that was developed by the Tobacco Control arm of the National Cancer Institute and has been used in multiple studies of smoking cessation apps [27,29-31]. QuitGuide helps users formulate a plan for quitting and provides educational resources with information helpful for quitting such as the medications for smoking cessation approved by the Food and Drug Administration. It has functions such as tracking cravings and triggers and can enable social connections. QuitGuide is publicly available for free [32]. Interviews lasted for an average of an hour each, were audio-recorded over Zoom, and augmented with note taking. Audio data were professionally transcribed.

### Participants

Participants were current or former smokers who self-identified as Black or African American adults 18 years and older, with English proficiency and ownership of a smartphone, iPad, or tablet. Current smokers were defined as those who smoked at least 100 cigarettes or used other nicotine products in their entire lifetime and still smoked. Former smokers were defined as those who smoked up to 100 cigarettes in their lifetime but had not smoked in the past 30 days. Smokers were purposefully recruited to reflect diverse smoking habits including the use of electronic cigarettes and other tobacco products. Individuals who were not proficient in English, who did not identify as Black, who only smoked occasionally (but not at least 100 cigarettes in their lifetime) or had not used other nicotine products, who were not interested in technology use for smoking cessation, or who lacked ownership of a smartphone, or iPad, or tablet were excluded from participating in the study.

A total of 1083 individuals were contacted via the web platforms. A total of 48 respondents completed the initial screener with 47 meeting eligibility requirements. Of these, 27 participants gave consent and were scheduled for interviews, whereas only 18 Black participants completed interviews. The other eligible participants did not show up on the day of the interview after reminders were sent and could not be reached via phone by the interviewer.

### Ethics Approval

The University of North Carolina at Chapel Hill institutional review board approved this study (approval number 20-1564). All participants provided informed consent via a telephone conversation, consent was confirmed right before their interviews, and the data were deidentified afterward. After completion of the interviews, participants were each emailed a US $50 incentive gift card and additional resources related to smoking cessation, as requested.

### Survey and Interview

In the preinterview REDCap survey, we asked questions about demographics, smoking history or habits, and cellular phone usage. We collected information on age, gender, race, history of tobacco use, current tobacco product use, history of quit attempts, and attempts with alternative approaches and willingness to quit. Using an in-depth, open-ended interview guide developed by the research team, participants provided feedback on their experiences with different features of QuitGuide after exploring the app on their smartphones for at least 1 week. Participants responded to a series of questions related to their thoughts about the app’s messages, the way those messages were presented, the functionality of the app, connecting with others through the app, and how likely they were to recommend the app to a family member or friend. We also asked about their preferences for an ideal smoking cessation app for future development. Depending on the discussion, participants could offer suggestions unprompted or in response to a more direct question or probe.

### Analysis

A coding-based content analysis approach identified themes within and across the content of interviews, following rigorous methods [33]. First, members of the team (CE, LML, and MEG) created a common codebook with coding categories derived from the interview guide questions. Interview transcripts were uploaded into the NVivo 12 (QRS International) qualitative software for analysis. Two members of the analytic team (LML and MEG) piloted the codebook by independently dual-coding 4 interview transcripts. Each analyst independently read and deductively coded these transcripts and met to reach consensus to further refine the codebook, adding additional codes as needed. Once the codebook’s definitions and decision rules were fine-tuned and finalized, one coder applied the codebook to the remaining set of interview transcripts. The other analyst then reviewed the coding, and the qualitative team met to reconcile any discrepancies by consensus. The data were further analyzed by creating and reviewing code and code concurrence reports and organizing the data within code reports by category, extracting supporting and salient quotes. The analysis focused on suggestions, preferences, and considerations related to developing a future app for smoking cessation. Because the sample size of qualitative studies is an iterative process and determined when thematic saturation is reached, the study team determined that the saturation of common themes was reached upon completion of 18 interviews [34].

## Results

### Demographic and Smoking History of Participants

Participants were aged 51.7 years on average, and most of them (n=14) were women and current smokers (Table 1).

All participants had used combustible tobacco, and 10 of the 14 current smokers reported quit attempts within the past year (Table 2).

**Table 1:** Demographic characteristics (N=18).

Characteristic		Value
**Gender, n (%)**		
	Female	14 (78)
Age (years), mean (SD)		51.7 (9.9)
**Age category (years), n (%)**		
	18-24	0 (0)
	25-34	1 (6)
	35-44	4 (22)
	45-54	3 (17)
	55-64	8 (44)
	>65	2 (11)
**Race, n (%)**		
	Black or African American	18 (100)
**Education, n (%)**		
	Less than high school	0 (0)
	High school diploma or GED^a^	6 (33)
	Associate degree or some college	8 (44)
	Bachelor’s degree	1 (6)
	Master’s degree	3 (17)
**Employment status, n (%)**		
	Employed or self-employed	10 (56)
	Unemployed	8 (44)
**Annual household income per year (US$) (n=17), n (%)**		
	0-29,999	8 (47)
	30,000-59,999	6 (35)
	60,000 or more	3 (18)

^a^GED: general educational development.

**Table 2:** Smoking history (N=18).

Smoking history			Value
**Age first started smoking (years), n (%)**			
	10-17	7 (39)	
	18-24	10 (56)	
	>25	1 (6)	
**Current smoker, n (%)**			
	Yes	14 (78)	
	No (former smoker)	4 (22)	
**Cigarettes smoked per day, n (%)**			
	0 (former smoker)	4 (22)	
	1-10	6 (33)	
	11-20	7 (39)	
	21-30	1 (6)	
	>31	0 (0)	
**Minutes to first cigarette upon waking, n (%)**			
	N/A^a^ (former smoker)	4 (22)	
	<5	4 (22)	
	6-30	8 (44)	
	31-60	2 (11)	
	>60	0 (0)	
**Quit attempts (current smokers) (n=14), n (%)**			
	Yes	10 (71)	
	No	4 (29)	
**Quit attempts within last 12 months (current smokers) (n=10), n (%)**			
	1-3 times	10 (100)	
	3-6 times	0 (0)	
	>6 times	0 (0)	
**Tobacco products used, n (%)**			
	Combustible cigarettes	18 (100)	
**History of electronic cigarettes used (n=14), n (%)**			
	None	10 (56)	
	Yes	8 (44)	
**Other nicotine products used (n=14), n (%)**			
	None	10 (71)	
	Other (patches, nicotrol inhalation system, and nicotine gum such as Nicorette)	4 (29)	

^a^N/A: not applicable.

### Major Themes

The team examined themes within five major areas relevant for developing a future mHealth smoking cessation app: (1) content needs, (2) format needs, (3) functionality, (4) social network, and (5) the need for inclusivity for Black individuals (Textbox 1).

**Mobile health app major themes from participants and description.**

**Content needs**

- Focus of the type of information in the app

**Format needs**

- Style of delivering information

**Functionality**

- Physical features such as tracking, information entry, reminders, and ability to personalize the app

**Social network**

- Connection with social groups

**Inclusivity**

- Creating an app designed specifically to appeal to and cater to the needs of Black smokers

### Content Needs

Although many participants endorsed the idea of seeing information about the negative effects of smoking, a few noted that it would not be helpful because such information is well known or that it would be more helpful for younger people who may be just starting smoking. In addition, many felt that it would be helpful to see content about the benefits of quitting, particularly health and other body-related benefits (ie, improved complexion), or financial benefits.

…Maybe some information about short- and long-term benefit like if I could not smoke for five days, then my lungs may be clearer, and I may not cough as much. To just really, be specific where possible about what the benefits could be.

65-year-old female and former smoker

Participants also felt that it would be helpful for the app to contain stories or testimonials from other people who have quit smoking or are trying to quit smoking, noting that these stories could increase engagement, motivate users, offer ideas for quit strategies, and reduce feelings of isolation.

I just wouldn’t mind knowin’ about other people’s experience, how they—why they started and all that, and how people quit, just testimonies or something on how people quit. Maybe they know something I don’t know or haven’t tried.

65-year-old female and current smoker

The majority of participants felt that it would be helpful if the app contained strategies for quitting or cutting back smoking, including strategies for resisting cravings and replacing smoking with healthy alternatives to cope with stress. Several participants also noted that it would be helpful to offer strategies for quitting smoking gradually (in contrast to a cold turkey approach), and many participants felt that it would be helpful for the app to provide information on nicotine replacement therapy resources, with a few participants noting that the app would likely be more effective if used in conjunction with nicotine replacement therapy.

It's real easy to get discouraged because, like I said, smokin' is just like my second hand at work. I'm just so used to doin' them before I know it, I've done it. I couldn't do the patches, didn't try the gum or the lozenges. That might be a good thing to do along with this app. I think it would be very effective.

60-year-old female and current smoker

### Format Needs

Some participants provided feedback on the formats in which they would like to receive or interact with content or messages on an app, including use of images, notifications, and website links. Several noted that they would like to see video or images and, in particular, images related to the harms of smoking (eg, smokers’ lungs). Many participants wanted the format to include SMS text messaging or pop-up notifications in the app, and a few participants pointed to the benefits of active learning, such as “gamifying” the app to allow people to interact with information or allowing people to respond to articles and other users. Finally, several participants felt that it would be helpful for the app to provide links to additional resources or cessation support programs.

I think something that would be helpful too to maybe scare people and make ’em concentrate more on it: show pictures of lungs, smokers’ lungs. That’s very, very graphic. If you’ve ever seen pictures of it, it would make you think—because it doesn’t look good.

63-year-old male and current smoker

I think people like rewards. Maybe you give them a little bit and they have to answer a question. Then you give them a little bit more. I know maybe it feels gamey or gimmicky, but I just think that people like bite-sized information.

56-year-old female and former smoker

### Functionality

Most participants suggested including features that allow users to track smoking behavior including number of cigarettes smoked, times when smoking occurred (or time in between smoking), smoking locations, urges, cravings, and triggers. In addition, several suggested tracking moods, and one participant suggested tracking physical symptoms. Some participants noted that a tracking function could help with recognizing and reflecting on smoking patterns. Some participants also felt that a journal feature would be helpful in a future app (and others discussed liking or finding the journal feature in the QuitGuide app beneficial).

To see how many cigarettes you smoke and the times and how much time between each cigarette, that would be great…. I would be pushing for more time without smoking. I would want, say I smoke two cigarettes in an hour, I will wanna say maybe one cigarette in two hours. I would be trying to push myself.

55-year-old female and current smoker

Many participants felt that it would be helpful for an app to provide tailored feedback based on the information provided by the user through tracking or to provide feedback on the negative financial and health effects of smoking such as dollars saved versus spent buying cigarettes. Several participants highlighted the importance of acknowledging cessation success and offering positive reinforcement or “prizes” (eg, points or visual rewards like trophies) through the app.

I thought would be useful for chronic smokers like my spouse if you could see how much money you were spending if you smoke a pack of cigarettes a day.

65-year-old female and former smoker

I'd like maybe somethin' to be added that would really give me a boost if I did something well, like if I went all day without smoking… People love to be patted on the back.

60-year-old female and current smoker

In addition, many participants suggested that the app should proactively reach out to the user through notification prompts, checking in with the user or reminding them of strategies or their reasons for quitting. A few also noted the importance of being able to personalize prompts or choose the frequency or time of day that these reminders will be sent.

I think definitely being able to have a daily reminder or a constant reminder of your goal or your reason for quitting smoking… I think daily positive reminders, positive input, positive notes to keep you on a positive state a mind.

47-year-old female and current smoker

Just saying, We noticed that you smoked this amount, or you smoked heavier at this time. Would you like a message maybe 30 minutes before or an hour?

56-year-old female and former smoker

With regard to information entry, preferences varied. The majority preferred multiple-choice options, noting that it can be easier, faster, or more convenient to choose from available options. Some participants preferred typing a response, and a few preferred an app with options to enter information via multiple choice or by typing, or by offering an “other” option that users can fill in. Some participants felt comfortable with providing general information in an app, but many others noted that there were limits on the type of information they would be willing to provide (eg, not their social security number). Some were not comfortable with a location-tracking feature (eg, GPS) or suggested that it be an optional feature.

Sometimes, you can't really explain what you're feelin' sometimes. Multiple choice gives you a direction.

32-year-old female and current smoker

I would say a mix of the two ’cause—especially on a smartphone sometimes, having to type a lot of stuff is annoying… other times, if you don’t have a choice that fits you, it can deter you from wanting to use the thing if… it’s not flexible enough.

46-year-old female and current smoker

Many participants felt that it would be helpful to be able to personalize aspects of the app, such as entering their own motivation for quitting or uploading visuals about these reasons, uploading a profile photo, adding a motivational song or videos, or setting their own quit date.

I would definitely have a visual of your goal for quitting or your reasons for quitting, the steps…that you’ve taken in the past and that you’ve taken now, what has worked, what is working, what hasn’t worked… Sometimes, like I said, having a visual, you can mentally or internally say, “Well, this is why I’m quitting.” To actually see these things visually, I think that helps a lot.

47-year-old female and current smoker

### Social Network

Many participants felt that it would be helpful for the app to include messages from their friends and family encouraging them or reminding them not to smoke, and a few noted that it could be helpful to share information from the app with their loved ones (eg, having a “family quit plan” where multiple users can support one another). However, a few felt that it may not be helpful to engage friends and family in their app usage (or that it would depend on the person), expressing concerns about disappointing others if they were unsuccessful, being judged for smoking habits, or receiving unhelpful comments.

Usually, when I make a decision, it’s a personal commitment, and I don’t necessarily find it’s helpful for someone to say, “Oh, I thought you quit smoking,” or “Don’t smoke that cigarette.” There’s always that slippery slope about friends and family. Sometimes, your help isn’t helpful. As a matter of fact, it’s downright just the opposite. That’s very personal. It depends.

65-year-old female and former smoker

Many participants were interested in connecting or interacting with other users on the app (eg, being able to exchange feedback, encouragement, and support) or through the app (eg, connecting them with a “quit buddy” they could meet up with in person or talk to over the phone and offering support groups). At the same time, some pointed out that the value of connecting with other users will depend on the user, and that it would be helpful to allow users to personalize the ways in which they connect with others (eg, adding “friends” in the app and having security or privacy features).

When you have other people who know what you’re going through, understand what you’re going through, it helps to exchange that. It helps to talk about it because they understand where you’re coming from, how you’re feeling, because they felt the same thing, but they may have tried a different way to deal with it.

65-year-old female and current smoker

You would have to make that choice yourself if you wanna make your information public to other people who are using the app. Then, there would have to be some sorta security measures so that whoever’s on the app, they’ve been verified that they are only using that information to interact with other users of the app.

47-year-old female and current smoker

Furthermore, some felt that it would be helpful for an app to facilitate connections to a quit coach or sponsor (ie, someone who has already quit smoking and who can provide support). Preferences for linking the app to social media platforms (eg, Facebook) varied. Some participants were interested in the idea of the app connecting to social media platforms, and some were not interested at all.

### Inclusivity

In addition to preferences related to smoking cessation apps in general, participants were asked about their thoughts about designing an app that is more inclusive and appealing to Black smokers specifically. Some expressed concerns about the implications of such an app. Participants expressed concerns that a tailored app may exclude other racial groups, users may feel targeted, or the focus on this specific population may be reflective of stereotypes rather than recognizing factors that contribute to the smoking in this population such as stress. However, participants also provided feedback on ways that an app could be designed to be inclusive to this population, such as including images of Black people or diverse imagery in general.

Making sure that you have people that look like us in it… Most apps or most things now that we see, it’s normally a white person. If you see a person of color that looks like you, you’re more apt to listen.

57-year-old female and former smoker

Many participants also discussed the types of content that would be helpful to include to make an app more inclusive to Black individuals, including statistics or health information pertaining to Black individuals and information about how tobacco advertising may disproportionately target this population (Textbox 2).

**Theme and quotes.**

**Inclusivity**

- “I would certainly hope that an app that is geared towards Black people would speak to the health benefits and the health consequences as it relates to our demographic. We are more prone to high blood pressure. How does smoking impact that? We are under an enormous amount of stress… smoking certainly doesn't help that dynamic” (56-year-old female and former smoker)
- “I think really addressing the mental health aspects because the African American community tends to be the less likely to reach out for help with mental health issues or discuss mental health issues. Talkin’ about that as it relates to smoking might be helpful…. I think provide resources for mental health. Especially if you have numbers to—anonymous sources where people can talk or whatever, or chat groups or something” (37-year-old female and current smoker)
- “…it looks like the tobacco companies spend a lot more money and a lot more time and effort on marketing their products to the Black neighborhood than they do marketing their products in the white neighborhoods. See, now, white neighborhoods is, you can't have it on TV no more. They don't put them ads in magazines no more. The only place they can advertise is at the store where they sell the cigarettes at, and they do very little of that….” (56-year-old male and current smoker)
- “… Maybe showing about the cigarettes ads that you see. I live in an exclusive Afro-American community here, and there’s ads everywhere. You know, where if I go on the other side of town, where it’s a mixed, or maybe more White, there’s not as many ads…. It’s like you’re targeting Afro-Americans. You know, maybe show people how that’s being done, and, you know, because I’m pretty sure that’s influencing some youngsters to smoke because they think it’s cool….” (63-year-old male and current smoker)

One participant discussed how including stories and testimonials from others could help create an inclusive app, also noting that it could be helpful to hear from success stories from notable individuals (eg, Barak Obama and Black celebrities). Finally, a few participants noted the importance of including culturally relevant messaging (eg, referring to specific types of cigarettes brands commonly used within this population).

We are encouraged by our success stories in our community. Incorporating those somehow even if it's words, even if you didn't get the person to actually speak to it but incorporating that into it.

56-year-old female and former smoker

Well, different kind of cigarettes 'cause most Black people just smoke Newports. I started smokin' Mavericks, and it's a difference. They seem lighter, even though the same company makes 'em, and they got full flavor and non-menthol, all that stuff, different types of cigarettes that bring your cravin' down, other types.

32-year-old female and current smoker

Most Black people, African Americans, they’re gonna smoke a certain brand: Salem, Kool, Newport… Give a bad thing about the cigarette that I’m smokin’ not just the tobacco. Make it more personal…. Make it, ‘Hey, man, put that Newport down.

59-year-old male and current smoker

## Discussion

### Principal Findings

This study investigated the preferences of Black smokers for features of mHealth apps for smoking cessation, using QuitGuide as a reference. We found that Black smokers prefer specific features of content, format, functionality, social network, and the inclusivity of an app. Some of these preferences are similar to those already identified by the general population based on findings from previous studies, whereas feedback on increasing the inclusivity of the app is more specific to Black smokers. This study provides guidance for researchers on content, functionality, and other app characteristics to consider in the development of an app for smoking cessation to increase uptake and engagement among Black smokers.

### Comparison With Previous Work

Preferences for content needs covered a broad range of topics with some of them reflective of the need for increased smoking cessation education and services for Black smokers. In this study, many participants indicated a preference for content about the health and financial benefits of quitting, tips and recommendations for quitting, and topics that are usually covered in smoking cessation programs or by health care providers. Unfortunately, Black smokers are less likely to be offered cessation education or services compared with Whites [10] and will benefit from having such information in mHealth apps. The preference for inclusion of testimonials of others from similar backgrounds who were successful in quitting is consistent with previous studies, which show that smokers are generally interested in hearing about the quitting experiences of others [35]. One study also found that smokers are highly interested in content about the health and financial benefits of quitting [36]. In this study, some participants mentioned the need for an app to provide alternatives for cravings, which is similar to findings from other studies [37]. The areas mentioned under content preferences may be said to be reflective of the need for increased smoking cessation education and services for Black smokers.

Participants also suggested different formats of relaying the content, including videos or images, SMS text messaging, gamifying, and links to additional resources. Previous studies suggest that other formats of content besides text alone are more appealing, such as audio or information delivery in short excerpts instead of big blocks of texts [38].

We also found functionality that allows users to track progress and provide personalized motivation within an app was emphasized as part of what makes an app appealing. Previous studies show that the ability to adapt features to personal needs is rated as important [35,39,40]. Oliver et al [36] in a study to understand what smokers want in an mHealth app found that an assessment for their reason for smoking and creating a personalized plan based on their reason for smoking was highly preferred [38]. In another study among young adult smokers, Gowarty et al [26] found that participants indicated the need for a smoking cessation app to have motivational information and tracking features. Unfortunately, most smoking cessation apps lack these important aspects of functionality. In 2019, Vilardaga et al [41] found that up to 70% of smoking cessation apps had a tracking function but only 42% of apps provided personalized feedback and 36% of apps had a reward system. Similar to findings in this study, the appeal for financial rewards, prizes, and badges to reward progress in mHealth apps has been cited by many other studies [26,35,42]. Participants in this study were not keen about an app with location-tracking feature. Studies have shown that location-tracking concerns among Black individuals are due to the potential for legal consequences [43]. This concern may be a result of the long history of overpolicing or surveillance in Black communities.

Similar to our study, past studies have also indicated mixed findings about the social aspect of smoking cessation apps. Some studies have shown that smokers are typically interested in sharing the smoking cessation journey with family and friends as well as connecting with others through the app or on social media [26,35,39]. Other studies suggest that connections via social media are not as appealing [36,40]. Reasons for these mixed results may include discomfort with sharing health information with strangers via a web platform, discomfort with sharing one’s failure in the event of a relapse, and the social stigma attached to smoking [38,44]. In one study, participants suggested that a forum on social media where members can interact anonymously may be more appealing [40].

Inclusivity of the app for Black smokers was another major area in our study. The preference for having information on people with similar cultural backgrounds on mHealth apps for various health conditions is well established. In a study to develop an app for self-management of anxiety and depression for Black women, participants recommended that the ability to create a list of Black female therapists be included in the app [45]. Another study that described the development of an mHealth app for the promotion of physical activity in Black men reported that participants preferred that the app contain images of Black men as well as a physical activity plan for health conditions common among Black men [46]. Usability testing of the developed app in the same study showed that features that were tailored to Black men were among the features identified as most favorable by participants [46]. Furthermore, other work demonstrates that smoking cessation efforts should reflect cultural needs specific to Black smokers for increased effectiveness, and these interventions can be adjusted to accommodate unique stressors and styles of coping, as well as elements that affect interactions of Black people with the health care system [47,48]. Our work adds to this literature by examining the importance of inclusivity in mHealth apps for smoking cessation.

Another major point raised in our study was the marketing strategy of the tobacco industry in Black communities, which has led to increased rates of point-of sale marketing compared with non-Black communities [49-51]. Providing information about targeted advertising in an mHealth app may elicit negative reactions in Black smokers and displeasure toward the tobacco industry. Yerger et al [52] explored how Black smokers respond to information about targeting advertisements in Black communities and found that such information led to discussions about the need to quit smoking and the need to warn the young ones about smoking. Such information on an mHealth app for smoking cessation may have antismoking effects on users. At the same time, it is important to be cognizant of concerns that participants raised about the development of a smoking cessation app for Black smokers specifically, such as the concern that this may be reflective of stereotypes or racism, and that potential users could feel targeted because of their race. This concern has been well described in the literature [53-55]. While being mindful of these concerns in app development, it is also well known that smoking cessation efforts that factor in the cultural needs of the target population have greater effectiveness than interventions that are developed for a broader group of users [56-58]. A focus on the need for an mHealth app that is inclusive of the features that appeal to Black smokers is important based on several recommendations on the need for more research to examine smoking interventions among smokers from minority groups [59-61]. The development of and communication around such an app must be sensitive to these concerns. Designing an app that is inclusive of, but not specific to, Black smokers may be a more well-received approach. Transparency, reassurance about concerns related to targeting as well as engagement or collaborations with the target population in research, and decision-making processes can be helpful [62,63].

### Limitations

Although our study’s strengths include participants’ exposure to an existing app, QuitGuide, for at least 1 week before their interviews, more time with the app would have allowed for a more comprehensive assessment. However, participants were able to develop opinions on various aspects of the app even within a short period. Also, there are numerous mHealth apps for smoking cessation with many different features. The use of one smoking cessation app as a reference limits the list of preferred features that participants can infer from their experiences. Additionally, although participants reported these preferences, it is unclear whether participants will use apps that incorporate their preferences much more than general apps. We are also cognizant of the fact that other factors such as marketing play a role in uptake. Lastly, we used a convenience sample in the study, with a majority of participants being female and middle aged and recruited from the southeastern United States, and we did not analyze for differences based on other demographic characteristics; hence findings may not be generalizable to the entire Black smoking population.

### Conclusions

This study is one of the first to document preferred features for a smoking cessation app by adult Black smokers. Our study shows that although some mHealth app features that have been rated highly by smokers in the general population are also highly preferred by Black smokers, the cultural relevance of the app is also particularly important to Black smokers. The cultural relevance of mHealth apps for smoking cessation needs to be considered when developing a smoking cessation app geared toward Black end users. Future studies can apply these preferences in the development of mHealth apps and explore the usability, acceptance, and effectiveness of such apps by Black smokers over other smoking cessation apps developed for the general population.

## Abbreviations

mHealth: mobile health
REDCap: Research Electronic Data Capture
UNC: University of North Carolina

## Data Availability

The data sets generated or analyzed during this study are available from the corresponding author on reasonable request.
